# Effects of Application of Recycled Chicken Manure and Spent Mushroom Substrate on Organic Matter, Acidity, and Hydraulic Properties of Sandy Soils

**DOI:** 10.3390/ma14144036

**Published:** 2021-07-19

**Authors:** Jerzy Lipiec, Bogusław Usowicz, Jerzy Kłopotek, Marcin Turski, Magdalena Frąc

**Affiliations:** 1 Institute of Agrophysics, Polish Academy of Sciences, Doświadczalna 4, 20-290 Lublin, Poland; b.usowicz@ipan.lublin.pl (B.U.); m.turski@ipan.lublin.pl (M.T.); m.frac@ipan.lublin.pl (M.F.); 2 Agricultural Farm, Trzebieszów-Kolonia 52A, 21-404 Trzebieszów, Poland; jklopotek@wp.pl

**Keywords:** organic amendments, soil organic matter, soil water retention, soil hydraulic conductivity, soil pH, coarse textured soils

## Abstract

Soil organic matter is a key resource base for agriculture. However, its content in cultivated soils is low and often decreases. This study aimed at examining the effects of long-term application of chicken manure (CM) and spent mushroom substrate (SMS) on organic matter accumulation, acidity, and hydraulic properties of soil. Two podzol soils with sandy texture in Podlasie Region (Poland) were enriched with recycled CM (10 Mg ha^−1^) and SMS (20 Mg ha^−1^), respectively, every 1–2 years for 20 years. The application of CM and SMS increased soil organic matter content at the depths of 0–20, 20–40, and 40–60 cm, especially at 0–20 cm (by 102–201%). The initial soil pH increased in the CM- and SMS-amended soil by 1.7–2.0 units and 1.0–1.2 units, respectively. Soil bulk density at comparable depths increased and decreased following the addition of CM and SMS, respectively. The addition of CM increased field water capacity (at –100 hPa) in the range from 45.8 to 117.8% depending on the depth within the 0–60 cm layer. In the case of the SMS addition, the value of the parameter was in the range of 42.4–48.5% at two depths within 0–40 cm. Depending on the depth, CM reduced the content of transmission pores (>50 µm) in the range from 46.3 to 82.3% and increased the level of residual pores (<0.5 µm) by 91.0–198.6%. SMS increased the content of residual pores at the successive depths by 121.8, 251.0, and 30.3% and decreased or increased the content of transmission and storage pores. Additionally, it significantly reduced the saturated hydraulic conductivity at two depths within 0–40 cm. The fitted unsaturated hydraulic conductivity at two depths within the 0–40 cm layer increased and decreased in the CM- and SMS-amended soils, respectively. The results provide a novel insight into the application of recycled organic materials to sequester soil organic matter and improve crop productivity by increasing soil water retention capacity and decreasing acidity. This is of particular importance in the case of the studied low-productivity sandy acidic soils that have to be used in agriculture due to limited global land resources and rising food demand.

## 1. Introduction

Soil organic matter (SOM) plays a central role in driving soil processes and functions and is a key resource base for agriculture. It contributes to improvement of soil structure, water retention, and quality [1,2,3,4]. Some types of soil organic matter can hold several times more water than its weight in water [5]. Despite the positive effects, the SOM content in cultivated soils is low and often decreases [6]. Almost half of European agricultural soils contain from 0 to 2% of organic matter [3,7]. As indicated by research, long-term soil disturbance by tillage can induce up to a ca. 50% loss of organic carbon from the plough layers compared to natural ecosystems (e.g., [8]). This is mostly due to disintegration of soil aggregates protecting physically organic matter [9,10], soil respiration [11], narrow crop rotation without green manure/cover crops and crop residue exclusion [12,13], and erosion [14,15]. The soil organic matter decline rates are increased by the boosted effect of warming related to climate change [3,6]. The overall amount of biomass and SOM decay is equivalent to up to 20% of global fossil fuel emissions [16,17]. Therefore, the decline in SOM has been identified as a major current environmental threat and a soil degradation element, whereas its high levels provide a solution for climate-smart soil management [18,19] and crop production [3].

To reverse previous losses or enhance soil carbon stocks, various already well-recognized management practices are recommended (e.g., [6,18]). However, new insights are still needed to select the most effective and sustainable practices. The developing circular (economy) systems inspire undertakings towards reusing and recycling waste organic materials to create a closed-loop system contributing to the reduction of pollution and gas emissions [20,21]. The utilisation of poultry (chicken) manure from waste disposal and spent mushroom substrate (SMS) remaining after harvesting mushrooms is widely recommended for beneficial recycling in agriculture to obtain completely natural nutrient and organic carbon cycles and to pursue long-term sustainability [21,22]. Both organic materials are valuable components of composts [22,23,24]. Due to their alkaline reactions, the materials can diminish soil acidity [24,25] and enhance nutrient availability [26]. Moreover, poultry manure and litter are a source of chemical energy for electricity and heat generation [27,28], and SMS is used as a substrate for other mushroom-forming fungi and in the production of biofuels and enzymes [21]. Although there are various ways for utilisation of both organic materials, their application in agriculture can be dominant, since the levels of other uses are relatively small [27,29].

The production of poultry manure, mostly by chicken, and spent mushroom substrate is still increasing. Global poultry meat production reached nearly 98 thousand metric tons in 2019, which was higher by 17.5% than in 2012. The production of eggs was 76.7 million metric tons in 2018 and increased by over 100% since 1990 [30]. Spent mushroom substrate (SMS) consists of different types of straw, corn cobs, peanut shells, or cotton seed hulls [21,31]. With the high organic matter content in the range of 40.7–86.9% on a dry weight basis [29,32] and available nutrients, including nitrogen and phosphorus, spent mushroom substrate can be an alternative soil amendment in conventional and organic farming systems [33,34]. Large quantities of SMS are generated in China, the USA, and several European countries, and they increase along with the production of edible mushrooms. Since 1978, global production of mushrooms has increased more than 30-fold [35]. Production of 1 kg of mushrooms generates about 5 kg of spent mushroom substrate [33].

Changes in the soil structure and pore size distribution in response to organic additions affect soil water retention (WRC) and hydraulic conductivity [36,37]. The soil hydraulic properties governing water storage and movement within the soil profile are essential for effective and adequate management of drainage and agricultural irrigation [38,39,40,41].

Addition of organic materials can be particularly advisable for sandy soils with low quality and productivity due to the low organic matter content and water retention capacity and the high permeability and acidity values. These soils are often used in agriculture to ensure global food security [42,43]. Across the globe, they cover around 900 million ha [44]. The aim of this study was to examine the effects of long-term application of chicken manure and spent mushroom substrate on organic matter accumulation, water retention, and hydraulic conductivity of sandy soils.

## 2. Materials and Method

### 2.1. Site Description

Two experimental sites were located in agricultural fields of two private agricultural farms on sandy soils in Trzebieszów (Podlasie Region, Poland The examined soils are classified as Podzols with Albic (Es) and Spodic (Bs) horizons in the soil profile (WRB, 2015). The sequence of the main horizon is Ap–Es–Bs. The Podzols formed from sand and sandy loams of glacial origin are a characteristic soil cover for the region [45]. More than 60% of the area is used for crop production. The investigations were conducted on two farms. Cultivated fields of one of the farms (N 51.990203; E 22.59253, Control N 51.981085; E 22.5902171) were treated with 10 t ha^−1^ of composted chicken manure (CM). In turn, 20 t ha^−1^ of spent mushroom substrate (SMS) were applied on the field of the other farm (N 51.994215; E 22.55112, Control N 51.9970984; E 22.5477854). Both amendments were applied every 1–2 years in a 20-year period. In the Podlasie region, the concentration of poultry production and mushroom cultivation require recycling of large amounts of by-products. As indicated by the literature review, the contents of organic carbon in CM and SMS vary from 349 to 388 g kg^−1^ d.m. [22,46] and from 233 to 483 g kg^−1^ [47], respectively. The corresponding pH ranges are 7.0–8.8 [22]. Standard mineral fertilization and farmyard manure (every 4 years) were applied on the control fields. The crop rotation in both sites during last 20-year period included rape, wheat, maize, and triticale.

### 2.2. Soil Sampling and Measurements

Undisturbed and bulk soil samples were taken from 0–20, 20–40, and 40–60 cm depths in 2019. Undisturbed samples (100 cm^3^) were collected in steel cylinders (5.0 cm height) for laboratory measurements of the soil water retention curve (WRC) and bulk density in four replications from every field and depth. Soil samples were taken just after harvest of cereals and before tillage in both agricultural farms. This sampling scheme allowed discrimination between the effects of the organic amendments on the soil properties and those of the tillage operations used to prepare the seedbed. The WRC was determined using pressure plates (Soil Moisture Equipment Corp., Santa Barbara CA, USA) according to Richards’ method [48]. After saturation, the following suctions were successively applied to establish matric potentials: −70, −100, −158, −1000, −5000, and −15,500 hPa to obtain the water retention curve. The WRC was used to estimate the volume of transmission pores (>50 μm), storage pores (50–0.5 μm), and residual pores, as well as the bending space (<0.5 μm) according to the pore classification proposed by Greenland [49]. Additionally, field water capacity defined as the equilibrium volumetric soil water content at −100 hPa matric potential (pF 2.0) was derived from the water retention curve.

The measured soil water retention at the selected matric potentials was used to fit the soil water retention model proposed by van Genuchten [50]:

$$\theta \left(h\right)=\theta_{r}+\frac{\theta_{s}-\theta_{r}}{{\left[1+{\left(\alpha h\right)}^{n}\right]}^{1-1/n}}$$

where *θ_s_* = saturated water content (cm^3^ cm^−3^), *θ_r_* = residual water content (cm^3^ cm^−3^), *h* = matric potential (–cm), and *α* and *n* = fitting parameters that determine the shape of the soil water retention curve (*α* has units of 1/cm and is related to the bubbling pressure, whereas *n* is dimensionless).

The saturated hydraulic conductivity (SHC) [51] and bulk density (BD) as the ratio of the mass of soil dried at 105 °C to the initial soil volume of 100 cm^3^ [52] were determined in the 100 cm^3^ undisturbed soil samples (in 4 replicates).

The van Genuchten–Mualem equation [53] was used to describe the unsaturated hydraulic conductivity function:

$$K\left(h\right)=\frac{K_{s}{\left\{1\;-{\left(\alpha h\right)}^{mn}{\left[1\;+{\left(\alpha h\right)}^{n}\right]}^{-m}\right\}}^{2}}{{\left[1+{\left(\alpha h\right)}^{n}\right]}^{ml}}$$

where *K* (cm day^−1^) is hydraulic conductivity; *K_s_* (cm day^−1^) is saturated hydraulic conductivity; α, *m*, *n* and *l* are equation parameters; *m* = 1 − 1/*n*; and *l* is an empirical pore-connectivity parameter established at 0.5 [53].

From 10 to 15 bulk soil samples were collected randomly from the depths of 0–20, 20–40, and 40–60 cm and then mixed to obtain representative samples for each treatment. The samples were used to determine particle-size distribution with the laser-diffraction method, organic carbon content with the Tiurin titration method [54] converted to organic matter by multiplying by 1.72, and pH using a potentiometric meter.

The penetration resistance was measured using a penetrologger (Eijkelkamp) and a cone with a base area of 1 cm^2^ and an angle of 60°. The penetrologger was pressed into the soil by hand to a depth of 20 cm at a mean velocity of 2 cm s^−1^. The penetration resistance was recorded at a 1 cm depth. Ten measurements were conducted at each treatment.

## 3. Results

### 3.1. Basic Soil Properties

The organic amended vs. control soils in both fields had slightly higher contents of silt and lower amounts of sand (Table 1). Irrespective of the treatment, the soil with CM compared to the SMS application had slightly higher amounts of clay and lower levels of silt and sand. The penetration resistance of the spent mushroom substrate-amended soil (at 0–20 cm) was lower by 41% compared to the control (2.76 MPa) and did not differ between the chicken manure-amended and control soils (1.87–1.88 MPa). Depending on the depth, the particle density decreased by 0.06–0.07 Mg m^−3^ and 0.07–0.14 Mg m^−3^ in the CM- and SMS-amended soils, respectively.

### 3.2. Soil Organic Matter and Reaction (pH)

**Figure 1:**
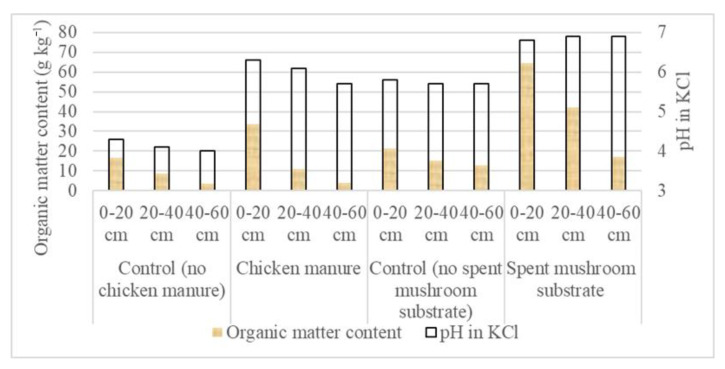
Soil organic matter content (yellow part of the bars) and pH (full length of the bars) of the control and chicken manure- and spent mushroom substrate-amended soils at depths of 0–20, 20–40, and 40–60 cm.

The long-term application of CM increased the initial soil organic matter (SOM) contents of 16.7, 8.6, and 3.4 (g kg^−1^) at depths of 0–20, 20–40, and 40–60 cm by 102.3, 27.9, and 17.6%, respectively (Figure 1). In the case of the SMS application, the initial SOM contents of 21.4, 15.0, and 12.8 g kg^−1^ at the successive depths increased by 201.2, 181.3, and 34.4%, respectively. The application of CM increased the initial soil pH (4.0–4.3) by 1.7–2.0 units, and the SMS amendment increased the initial values (5.7–5.8) by 1.0–1.2 units, depending on depth.

### 3.3. Soil Hydraulic Properties

**Figure 2:**
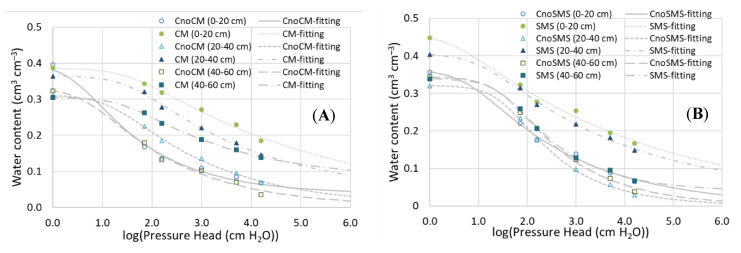
Fitted and measured soil water retention curves at depths of 0–20, 20–40, and 40–60 cm. CM—chicken manure (**A**), SMS—spent mushroom substrate (**B**), CnoCM—control for the CM-amended soil, CnoSMS—control for the SMS-amended soil substrate (SMS).

The addition of both organic materials resulted in considerably higher soil water contents measured at the same pressure head range (from −71 cm (1.85 log_10_(|–cm H_2_O|)) to 15,000 cm ~4.2 log_10_(|–cm H_2_O|) at all three comparable depths. An exception was the 40–60 cm in the SMS addition variant, where the differences were small and inconsistent in the pressure head range (Figure 2A,B). The most pronounced effect was observed at a depth of 0–20 cm, where the water content in the CM-amended vs. control soil increased by about 100–150%, depending on the pressure head range. The corresponding increases in the SMS-amended soil varied from approximately 60 to 115%. Comparison of the data in the soil profile indicates that, irrespective of the organic material type, the relative differences in the soil water contents within this pressure head range between the depths were greater in the amended than control soils.

The high coefficients of determination (R^2^ > 0.989) showed that the van Genuchten soil water retention model fitted the measured data very well (Table 2). The values of the shape parameter (*n*) indicated that the soil water retention curves were in general steeper in soils enriched with both organic materials (1.113–1.178) vs. the control soils (1.208–1.370), except in the depth of 40–60 cm in the SMS-amended soil with the higher *n* value (1.368) compared to the control. In the CM-amended soil, the fitted curves at all depths showed a longer flat pattern with more negative pressure heads at which air starts entering the soil matrix (1/scaling parameter α), i.e., 30.0–38.6 cm, depending on the depth, compared to 2.9–16.3 cm in the control soil. The addition of SMS, however, caused a decrease in the air-entry value at the two upper depths within 0–40 cm (from 6.3–37.2 cm to 2.7–11.2 cm) and an increase at a depth of 40–60 cm (from 34.7 to 38.0 cm).

As can be seen in Table 3, the addition of CM significantly reduced the content of transmission pores (>50 µm) at depths of 0–20, 20–40, and 40–60 cm by 82.3, 46.3, and 70.9%, respectively, and increased the content of residual pores (<0.5 µm) by 198.6, 91.0, and 156.7, respectively. The content of storage pores in the CM-amended soil increased by 20.0 and 7.1% at depths of 0–20 and 20–40 cm, respectively, and decreased by 8.9% at a depth of 40–60 cm. The decrease in the content of transmission pores at depths of 0–20 and 20–40 cm corresponds with the approximately three-fold lower saturated hydraulic conductivity (Ks) (15.8 vs. 4.7 m day^−1^ and 7.8 vs. 2.4 m day^−1^). The addition of SMS increased the content of residual pores (<0.5 µm) at the successive depths by 121.8, 251.0, and 30.3%, respectively. However, the contents of transmission and storage pores (>50–0.5 µm) decreased or increased depending on the depth, and the relative changes were much lower (from 5.7 to 25.6%) than in the case of residual pores. The addition of SMS caused a decrease in Ks within the depth of 0–40 cm, but the decline was relatively higher in the 20–40 cm layer (2.6 vs. 0.78 m/day) than 0–20 cm (5.7 vs. 4.7 m day^−1^).

The volumetric soil water content corresponding to field water capacity (at −100 hPa) at depths of 0–20 cm (15.2%), 20–40 cm (20.5%), and 40–60 cm (15.6%) in the control soil increased by 117.8, 45.8, and 58.3%, respectively, after the addition of chicken manure (Table 3). Correspondingly, the addition of the spent mushroom substrate increased the initial values of 19.9, 20.5, and 22.2% at the successive depths by 48.5, 42.4, and 1.9%, respectively. The increases in field water capacity after the addition of both organic materials, especially CM, were greater than those in water contents at saturation, as reflected by the higher values of the relative field capacity (ratio of field water capacity to saturated water content at pF 0) (data not shown).

**Figure 3:**
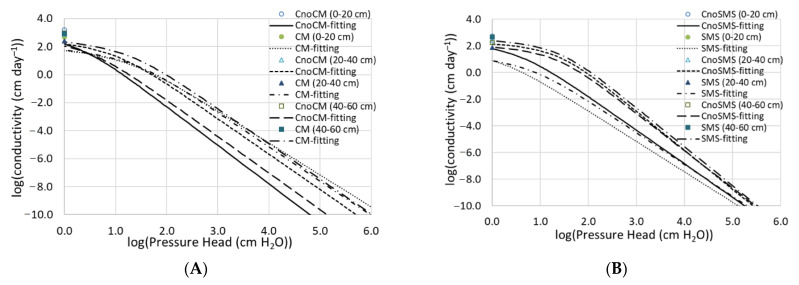
Fitted saturated and unsaturated hydraulic conductivity (as a function of the pressure head) at depths of 0–20, 20–40, and 40–60 cm. CM—chicken manure (**A**), SMS—spent mushroom substrate (**B**), CnoCM—control for the CM-amended soil, CnoSMS—control for the SMS-amended soil.

Both organic amendments influenced the pattern of the fitted hydraulic conductivity curve differently (Figure 3A,B). The CM addition resulted in higher unsaturated hydraulic conductivity (K) at depths of 0–20 and 40–60 cm across the whole pressure head range, with a greater increase at more negative pressure head values (Figure 3A). Compared to the control, the K value in the CM-amended soil at a depth of 20–40 cm was slightly higher in the high-pressure head section <1.5 log_10_(|–cm H_2_O|)) and slightly lower at more negative pressure heads. At depths of 0–20 and 40–60 cm, the greater K value in the CM-amended vs. control soil corresponded with the higher field water capacity, content of residual pores, and bending space <0.5 µm (Table 3). The addition of SMS, however, resulted in lower saturated and unsaturated hydraulic conductivity at both upper depths to 40 cm and slightly higher values of the parameter at a depth of 40–60 cm in the whole range of pressure heads (Figure 3B).

## 4. Discussion

### 4.1. Soil Organic Matter and Acidity

The application of the organic materials appreciably increased the soil organic matter content at all three depths within 0–60 cm. Noteworthily, the increase in soil organic matter in the top 0–20 cm of soil after the addition of both chicken manure and spent mushroom substrate (by 17.1–43.1 g kg^−1^) was substantially higher than after application of conservation tillage (by ~1.7–3.4 g kg^−1^) and mineral fertilizers alone (by ~5.1 g kg^−1^) [44] in similarly textured soils. These results demonstrate that the organic matter in the recycled materials applied, especially in the SMS variant, was in a relatively stable form to build-up soil organic matter pools. It is worth noting that also at a depth of 20–40 cm, the increase in soil organic matter (SOM) was greater after the application of SMS than CM. The more pronounced effect of SMS on soil organic matter accumulation up to the depth of 40 can be related to both the additional organic matter provided and the deeper tillage operations applied to the soil amended with SMS (to approx. 35 cm) than CM (to approx. 25 cm). The accumulation of SOM may result not only from the organic amendment materials applied but also from the increasing crop yield and associated crop residues [6,12]. Despite the significant increase, the content of SOM in the amended soils was still lower than that in the sandy Plaggen soils of late medieval origin in northwest Europe (approx. 112 g kg^−1^) produced by long-term additions of sod, litter, and manure to increase soil fertility [44]. This indicates that sandy soils have high potential for accumulation of soil organic matter and improvement through appropriate soil management practices. A more detailed analysis of soil organic matter quality has revealed that SMS application increases the share of valuable humic acids and the associated humic to fulvic acid ratio [55].

The increase in the soil organic matter content observed in this study is part of a global strategy to enhance soil resilience to climate change and ensure sustainable food and nutrition security (e.g., [18]). This strategy has been promoted recently by, e.g., the “4 per mille” initiative requesting to increase global carbon sequestration stocks by 4 g kg^−1^ soil organic carbon every year [56]. Furthermore, inorganic nitrogen compounds obtained by microbial degradation from organic nitrogen in both materials can be utilized for production of new cell material by many organisms and for plant growth. In this way, organic nitrogen and other nutrients are recycled within local environments [26,47,57] and, accordingly, reduce the use of costly chemical input-dependent manufactured fertilizers [3,6,47,58].

As can be seen in Figure 1, the addition of both CM and SMS increased initial soil pH in the topsoil by up to ca. two units. This is attributed to the alkaline reaction (pH > 7) of both organic materials (e.g., [24,25]. Therefore, raising the pH of the studied acidic soils has a beneficial effect on soil quality and crop production and contributes to reduction of the use of new carbonate rocks to neutralize soil acidity [59].

### 4.2. Hydraulic Properties

Our results showed that the addition of both organic materials significantly decreased and increased the content of transmission pores (>50 µm) and residual pores (<0.5 µm), respectively (Table 3). The increase in the content of residual inter-particle pores can be caused by the settling of small organic matter particles (coatings) on the coarse soil particles [2,60]. The settling is favoured by the considerably higher specific surface area of the organic amendments, e.g., 246 m^2^ g^−1^ for chicken manure [61], compared to that in the studied sandy soils (<35 m^2^ g^−1^) [62]. This explanation can be supported by the positive correlation between the surface area of organic biochars and the micro-pore volume of soil observed by Lehmann and Joseph [63], Villagra-Mendoza and Horn [2], and Gluba et al. [4]. The decrease in the content of large transmission pores (>50 µm) in the soils amended with both organic materials in our study was favourably reflected by the lower saturated hydraulic conductivity of the analysed permeable soils. Reduced content of transmission pores and increased field water capacity in response to organic additions are beneficial to water storage of sandy soils and resistance to increased drought frequency under climate change. As for storage pores (50–0.5 µm), the effect of the soil amendments was less pronounced than on transmission pores but differed between the CM and SMS and between soil depths. The pronounced increase in storage pores at depths of 0–20 and 20–40 cm in the CM-amended soil corresponded with high unsaturated hydraulic conductivity (at most ranges of pressure head) (Figure 3A, Table 3). In the SMS-amended soil, the most pronounced reduction of storage pores at a depth of 20–40 cm corresponded with reduction of unsaturated hydraulic conductivity over the whole range of the pressure head (Figure 3B). At the same time, the application of both organic materials caused a significant increase in residual pores and bending space (<0.5 µm) that hold plant-unavailable water. However, this adverse response had no negative effect on crop productivity, which was higher in the amended than reference fields, as indicated by the owners of the experimental farms (personal communication). Overall, the results of this study indicate that long-term application of recycled organic materials can be a suitable means to improve crop productivity and quality of coarse-textured soils by increasing water retention capacity and decreasing acidity. It is worth noting that addition of organic amendments to poorly drained fine-textured soils, loamy, or clay soils can improve soil structure, water penetration, aeration [64,65], and water retention in variously textured soils [66].

### 4.3. Suitability of Measurement and Calculated Methods for Determining Soil Porosity

**Figure 4:**
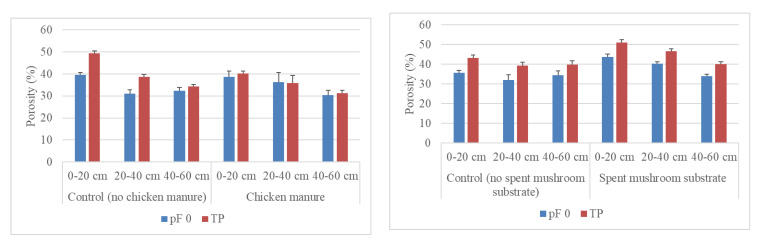
Porosity determined from water retention measurements (pF 0) and porosity calculated from measurements of bulk density and particle density (TP) at depths of 0–20, 20–40, and 40–60 cm. The bars represent standard deviations.

Our results showed that the degree of agreement between soil total porosities determined from the volume of water contained in a saturated sample (pF 0, i.e., log_10_(|1 cm H_2_O|)) and calculated from measurements of particle density and bulk density [67] differed depending on the type of the organic material applied. In the case of the CM-amended soil, the total porosities from both methods were similar at all depths (Figure 4).

**Figure 5:**
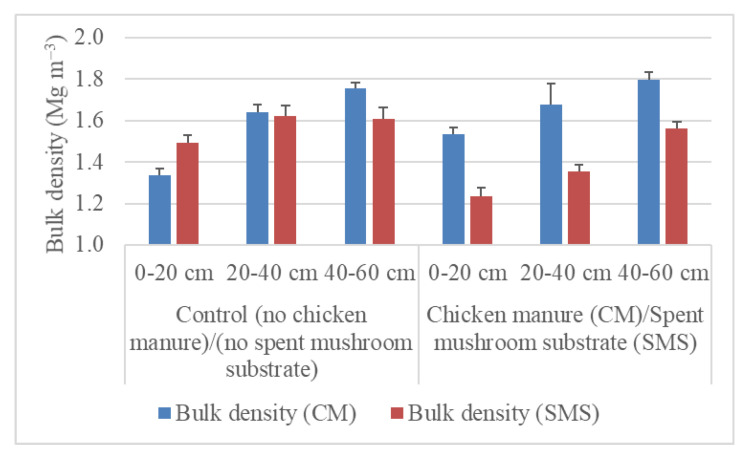
Bulk density at depths of 0–20, 20–40, and 40–60 cm. The bars represent standard deviations.

However, in the SMS-amended and both control soils, the total porosities derived from the volume of water in saturated soil compared to the calculated porosities were lower. This indicates that the water outflow by gravity during a few-second transfer of soil samples saturated with water to a weighing balance was lower in the CM-amended soil than in the other treatments. The lower water outflow in the CM-amended soil can be related to formation of a structure with a lower share of large pores draining out water by gravity and a greater share of small pores retaining water by capillary forces. This explanation can be supported by the highest water content maintained at field water capacity in the CM-amended soil at all depths (Table 3). The greater share of smaller pores in the CM than SMS treatment can be further supported by higher bulk density (Figure 5). This indicates that the different gravitational water outflow can be a source of inaccuracy in the determination of total soil porosity at pF 0 of soil enriched with different organic materials. Our comparison indicates that this inaccuracy can be alleviated by calculation of total porosity from particle and bulk densities without saturation of soil.

## 5. Conclusions

The results of this study allowed for the formulation of the following conclusions:

1. The long-term application of recycled chicken manure (CM) and spent mushroom substrate (SMS) increased the organic matter content and decreased acidity in sandy soils.
2. CM significantly reduced the content of transmission pores (>50 µm) and increased that of residual pores (<0.5 µm) and the water content corresponding to the field water capacity at all 3 depths within 0–60 cm. SMS significantly increased the content of residual pores and the water content corresponding to the field water capacity at two depths within 0–40 cm.
3. CM reduced the saturated hydraulic conductivity significantly at two depths within 0–40 cm and insignificantly at a depth of 40–60 cm. The insignificant reduction of the saturated hydraulic conductivity at all depths was caused by the addition of SMS. The application of CM and SMS decreased and increased the air entry values 1/α, respectively. The fitted unsaturated hydraulic conductivity at two depths within 0–40 cm increased and decreased in response to the CM and SMS application, respectively.
4. Long-term use of recycled organic materials can be a suitable means to improve the quality and crop productivity of sandy soils by increasing water retention capacity and decreasing acidity.

## Figures and Tables

**Table 1:** Soil textural composition and penetration resistance at depth 0–20 cm and particle density at depths 0–20, 20–40, and 40–60 cm. Standard errors are shown in brackets.

Treatments	Textural Fractions, g kg^−1^			Penetration Resistance	Particle Density
	Clay (<2 μm)	Silt (2–50 μm)	Sand (>50 μm)	(MPa)	(Mg m^−3^) 0–20, 20–40, 40–60 cm
Control (no chicken manure)	20.5	270.4	709.1	1.87 (0.11)	2.64, 2.67, 2.67
Chicken manure	21.6	345.9	632.5	1.88 (0.09)	2.57, 2.61, 2.62
Control (no spent mushroom substr.)	18.0	240.9	741.1	2.76 (0.10)	2.63, 2.67, 2.67
Spent mushroom substrate	19.4	269.1	711.5	1.63 (0.09)	2.53, 2.53, 2.60

**Table 2:** Fitted values of the residual water content (*θ_r_*), saturated water content (*θ_s_*), scaling parameter (*α*), and shape parameter (*n*) of the soil water retention model (van Genuchten, 1980).

Treatment	Depth (cm)	*θ_r_* (cm^3^ cm^−3^)	*θ_s_* (cm^3^ m^−3^)	*α* (cm^−1^)	1/*α* (cm)	*n*	R^2^
Control (no chicken manure)	0–20	0.036	0.400	0.3414	2.9	1.301	0.993
	20–40	0.000	0.312	0.0614	16.3	1.212	0.998
	40–60	0.000	0.331	0.1946	5.1	1.242	0.991
Chicken manure	0–20	0.001	0.387	0.0275	36.4	1.113	0.993
	20–40	0.035	0.366	0.0259	38.6	1.178	0.996
	40–60	0.076	0.306	0.0333	30.0	1.204	0.998
Control (no spent mushroom substrate)	0–20	0.000	0.363	0.1582	6.3	1.208	0.995
	20–40	0.000	0.321	0.0269	37.2	1.370	0.998
	40–60	0.000	0.344	0.0288	34.7	1.320	0.997
Spent mushroom substrate	0–20	0.000	0.461	0.3650	2.7	1.113	0.989
	20–40	0.030	0.406	0.0891	11.2	1.155	0.997
	40–60	0.039	0.339	0.0263	38.0	1.368	0.998

**Table 3:** Pore size distribution, field water capacity, and saturated hydraulic conductivities of the control and chicken manure- and spent mushroom substrate-amended soils at depths of 0–20, 20–40, and 40–60 cm. Standard deviations are shown in brackets. Means with different letters denote significant differences between the control and treatments at the same depth at the 5% level by the LSD test.

Treatments	Depth (cm)	Porosity (% *v*/*v*)			Field Water Capacity %, *v*/*v*	Saturated Hydraulic Conductivity m/day
		Transmission Pores >50 µm	Storage Pores 50–0.5 µm	Residual Pores and Bonding Space <0.5 µm		
Control (no chicken manure)	0–20	21.4 (1.26) a	11.0 (1.27) b	7.3 (1.50) c	15.2 (0.66) d	15.8 (4.51) a
	20–40	8.0 (1.48) a	14.0 (2.16) a	8.9 (1.27) b	20.5 (1.12) b	7.8 (3.80) a
	40–60	14.1 (2.70) a	12.1 (0.79) b	6.0 (2.86) b	15.6 (4.24) a	9.2 (4.02) a
Chicken manure	0–20	3.8 (1.25) c	13.2 (2.53) ab	21.8 (1.03) a	33.1 (2.38) a	4.7 (2.18) b
	20–40	4.3 (2.33) b	15.0 (4.29) a	17.0 (1.92) a	29.9 (2.10) a	2.4 (0.73) b
	40–60	4.1 (1.59) c	11.0 (1.00) b	15.4 (2.41) a	24.7 (2.71) c	8.5 (4.63) a
Control (no spent mushroom substrate)	0–20	13.2 (1.05) b	13.8 (0.21) a	8.7 (0.72) c	19.9 (0.86) c	5.7 (1.86) b
	20–40	8.0 (0.74) a	19.2 (1.24) a	4.9 (2.07) b	20.5 (2.86) b	2.6 (0.23) b
	40–60	8.1 (0.43) b	19.6 (1.33) a	6.6 (2.76) b	22.2 (1.83) b	1.8 (0.71) b
Spent mushroom substrate	0–20	11.3 (1.50) b	13.1 (0.80) a	19.3 (1.44) b	29.5 (1.06) b	4.7 (1.03) b
	20–40	8.8 (2.03) a	14.3 (1.64) a	17.2 (2.30) a	29.2 (2.10) b	0.78 (0.20) b
	40–60	7.3 (0.98) b	18.0 (1.11) a	8.6 (0.65) b	23.2 (0.28) bc	4.4 (0.83) ab

## Data Availability

The data underlying this article are available in Zenodo repository (https://zenodo.org/, accessed on 30 July 2021).
